# Meta-Analysis for Genome-Wide Association Study Identifies Multiple Variants at the BIN1 Locus Associated with Late-Onset Alzheimer's Disease

**DOI:** 10.1371/journal.pone.0016616

**Published:** 2011-02-24

**Authors:** Xiaolan Hu, Eve Pickering, Yingxue Cathy Liu, Stephanie Hall, Helene Fournier, Elyse Katz, Bryan Dechairo, Sally John, Paul Van Eerdewegh, Holly Soares, the Alzheimer's Disease Neuroimaging Initiative

**Affiliations:** 1 Molecular Medicine, Pfizer Inc., Groton, Connecticut, United States of America; 2 Research Statistics, Pfizer Inc., Groton, Connecticut, United States of America; 3 Clinical Statistics, Pfizer Inc., Shanghai, China; 4 Genizon Biosciences Inc, Montreal, Canada; 5 Translational Medicine, Pfizer Inc., Groton, Connecticut, United States of America; Mental Health Research Institute of Victoria, Australia

## Abstract

Recent GWAS studies focused on uncovering novel genetic loci related to AD have revealed associations with variants near *CLU*, *CR1*, *PICALM* and *BIN1*. In this study, we conducted a genome-wide association study in an independent set of 1034 cases and 1186 controls using the Illumina genotyping platforms. By coupling our data with available GWAS datasets from the ADNI and GenADA, we replicated the original associations in both *PICALM* (rs3851179) and *CR1* (rs3818361). The *PICALM* variant seems to be non-significant after we adjusted for *APOE e4* status. We further tested our top markers in 751 independent cases and 751 matched controls. Besides the markers close to the APOE locus, a marker (rs12989701) upstream of *BIN1* locus was replicated and the combined analysis reached genome-wide significance level (p = 5E-08). We combined our data with the published Harold et al. study and meta-analysis with all available 6521 cases and 10360 controls at the *BIN1* locus revealed two significant variants (rs12989701, p = 1.32E-10 and rs744373, p = 3.16E-10) in limited linkage disequilibrium (r^2^ = 0.05) with each other. The independent contribution of both SNPs was supported by haplotype conditional analysis. We also conducted multivariate analysis in canonical pathways and identified a consistent signal in the downstream pathways targeted by Gleevec (P = 0.004 in Pfizer; P = 0.028 in ADNI and P = 0.04 in GenADA). We further tested variants in *CLU*, *PICALM*, *BIN1* and *CR1* for association with disease progression in 597 AD patients where longitudinal cognitive measures are sufficient. Both the *PICALM* and *CLU* variants showed nominal significant association with cognitive decline as measured by change in Clinical Dementia Rating-sum of boxes (CDR-SB) score from the baseline but did not pass multiple-test correction. Future experiments will help us better understand potential roles of these genetic loci in AD pathology.

## Introduction

Alzheimer's disease (AD) is a neurodegenerative disease clinically characterized by memory impairment and pathologically characterized by the formation of amyloid plaques and neurofibrillary tangles in the brain. Less than 5% of AD patients can be categorized as early-onset disease (diagnosis before age 65). The cause for this subset of disease has been linked to gene mutations in *amyloid precursor protein* (*APP*), *presenilin 1* (*PSEN1*), *presenilin 2* (*PSEN2*) (reviewed in [1]) and duplications of *APP* [2]. The major form of AD, late-onset AD (LOAD), also has a strong genetic component. Large twin studies have estimated LOAD heritability ranging from 60 to 80 percent [3]. *APOE* is the primary genetic risk factor in LOAD [4].

The *APOE E4* variant does not account for all cases of AD. It is present in less than 50% in European AD cases and occurs even less frequently in African, Asian and Hispanic AD populations. Identification of additional genetic variants apart from *APOE* has been challenging due in part to the smaller effect sizes of these variants. Genome-wide association studies provide an unbiased approach to test the “common variants common disease” hypothesis. Previous GWAS studies [5]–[12] revealed promising candidates such as *GAB2* [6] and *PCDH11X* [10] but few have been independently replicated. Two recent large studies [13], [14] presented compelling genetic evidence for a common variant at the *CLU* locus to play a role in disease susceptibility. Each study discovered an additional locus near *PICALM* or *CR1* reached genome-wide significance level. In this study, we conducted a GWAS scan in 1034 cases and 1186 controls mostly collected from Pfizer clinical trials. We first examined genetic markers associated with disease susceptibility for late-onset AD by combining available GWAS data from Pfizer, Alzheimer's Disease NeuroImaging Initiative (ADNI) [11] and Genotype-Phenotype Alzheimer's disease Associations (GenADA) [8]. The top variants were further tested in an independent data set (751 cases and 751 controls). A pathway analysis was conducted to take into account the joint effects of multiple variants to complement the single variant analysis for disease susceptibility. We also investigated the association of the validated variants with disease progression in AD patients where longitudinal cognitive data are available.

## Results

### Genome-wide association studies on AD

To identify common genetic markers involved in AD susceptibility and progression, we first conducted a genome-wide association study in 1034 cases and 1186 controls (the re-matched analyzed set included 733 LOAD cases and 792 controls). To this initial data set, we added available genome-wide individual data from ADNI and GenADA to increase the statistical power (a total of 1831 AD cases and 1764 controls). All genotyping data were subjected to a strict quality control process including call rates, Hardy-Weinberg equilibrium (HWE) test, sample heterogeneity, gender check (samples with mismatched gender information from the genotype data and the reported gender information from the clinical database were removed from the analysis) and population stratification (only Caucasians were included in the analysis set). Since limited number of markers are shared between Affymetrix 550 K (GenADA) and Illumina HumanHap 550/610 platforms (Pfizer and ADNI), we imputed the GenADA data set to the non-singleton HapMap SNPs based on the HapMap III reference haplotypes in unrelated Caucasian individuals. Poorly imputed SNPs (r^2^ less than 0.3 or minor allele frequency less than 1%) were removed before any further analysis.

We examined association of single nucleotide polymorphisms with AD disease status (χ^2^ allelic test) in each cleaned case/control sample set using PLINK [15] (all summary statistics data associated with the Pfizer data set are listed in Table S1). No significant population stratification is present in any data set. The estimated inflation factor lambda, as a measure of population stratification, is 1.04, 1.02 and 1.00 in the Pfizer, ADNI and imputed GenADA sample sets respectively. We combined evidences from three cohorts using weighted z-score statistics [16]. In addition to markers adjacent to the *APOE* locus, meta-analysis revealed a number of distinct loci with suggestive association signals with p values less than 1×10^−6^ (Table 1). Furthermore, we replicated previously reported associations in *CR1* (rs3818361, P = 0.001, OR = 1.22) and *PICALM* (rs3851179, p = 0.006, OR = 0.87) loci. The direction of effect for both variants is consistent across each individual sample set (Table 2). In addition, the effect of the PICALM variant appears to be confounded by the APOE alleles despite this variant is located at a different chromosome. The variant is no longer significant after we adjust for APOE e4 status in the analysis (p = 0.26). The distribution of the *CLU* allele (rs11136000) is not significantly different in cases and controls. However, odds ratios for this variant appear to be consistent with the previous studies and close to be significant in the Pfizer sample set (P = 0.068, OR = 0.87).

**Table 1:** Top markers with P<0.000001 from GWAS study in 1831 AD cases and 1764 controls (Meta-analysis for Pfizer, ADNI and GenADA)^a^.

*CHR*	*Position (b.p.)*	*SNP*	*Allele 1*	*Allele 2*	*Combined P*	*Weighted Z-score* b
19	50087106	rs157580	G	A	2.79E-17	−8.46
19	50073874	rs6859	A	G	1.48E-10	6.41
19	50021054	rs10402271	G	T	1.47E-07	5.26
6	69672336	rs10485435	T	G	6.14E-07	4.99
6	70651135	rs2502562	A	G	1.57E-06	−4.80
19	49929652	rs2965101	C	T	2.09E-06	−4.74
1	216772136	rs4846486	A	C	2.33E-06	−4.72
19	49923318	rs2927488	A	G	2.86E-06	−4.68
5	71281720	rs1217745	T	C	3.24E-06	−4.66
2	127604455	rs12989701	A	C	4.68E-06	4.58
6	40917990	rs9369240	G	A	4.87E-06	4.57
3	59868076	rs624225	C	T	5.12E-06	−4.56
6	40909709	rs12664598	G	A	6.52E-06	4.51
5	7531914	rs252546	G	A	7.79E-06	−4.47
11	26651418	rs4551716	C	T	8.30E-06	−4.46
11	26619122	rs4497357	A	G	8.52E-06	4.45
3	59866016	rs643629	A	G	9.98E-06	−4.42

a Z-score was calculated after adjustment of genomic control in each sample set. Only SNPs present in all three data sets were included in the table.

b A negative Z-score indicates that Allele 1 is less frequent in cases and a positive Z-score indicates Allele 1 is more frequent in cases vs. controls.

**Table 2:** Association test results for previously identified variants in *CR1*, *PICALM* and *CLU* from three independent sample sets.

*SNP (Gene)*	*Allele 1*	*Allele 2*	*Data Set*	*# of Cases*	*# of Controls*	*MAF in Cases*	*MAF in Controls*	*P-value*	*Odds Ratio*
rs3818361^a^	A	G	Pfizer	733	792	0.217	0.195	0.136	1.143
(CR1)	A	G	ADNI	300	196	0.207	0.153	0.034	1.441
	A	G	GenADA^c^	798	776	0.214	0.18	0.019	1.234
			Combined	1831	1764	0.214	0.184	0.001	1.215
rs3851179^b^	A	G	Pfizer	732	792	0.327	0.367	0.018	0.835
(PICALM)	A	G	ADNI	300	196	0.343	0.37	0.392	0.891
	A	G	GenADA^c^	798	776	0.35	0.373	0.171	0.903
			Combined	1830	1764	0.339	0.37	0.006	0.872
rs11136000	A	G	Pfizer	733	791	0.393	0.425	0.068	0.874
(CLU)	A	G	ADNI	300	196	0.377	0.385	0.787	0.964
	A	G	GenADA^c^	798	776	0.352	0.356	0.806	0.982
			Combined	1831	1763	0.372	0.391	0.153	0.931

a The *CR1* variant remains borderline significant (P = 0.06) in logistic regression analysis adjusting for APOE e4 +/− status.

b The *PICALM* variant is no longer significant (P = 0.26) at the 0.05 level after we adjust for APOE e4 status (+/−).

c GenADA genotype data for the variants were imputed.

### Replication

**Figure 1:**
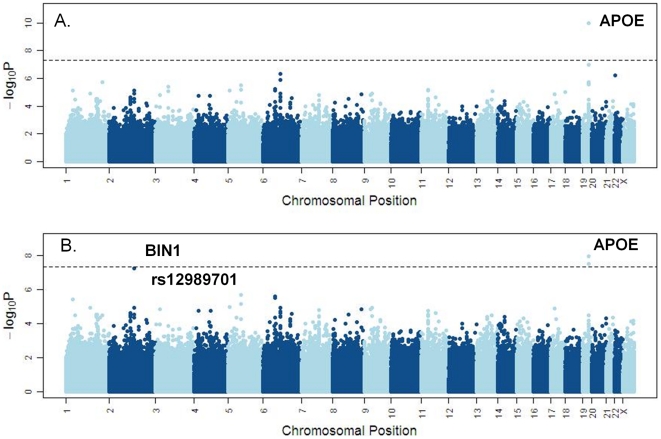
Manhattan plots for GWAS association meta-analysis results combining. **a) Pfizer, ADNI, GenADA; b) plus top marker results in the QFP replication set.** The line indicates genome wide significance level. Top markers at the APOE locus were removed in the plots to improve resolution for the other markers.

**Figure 2:**
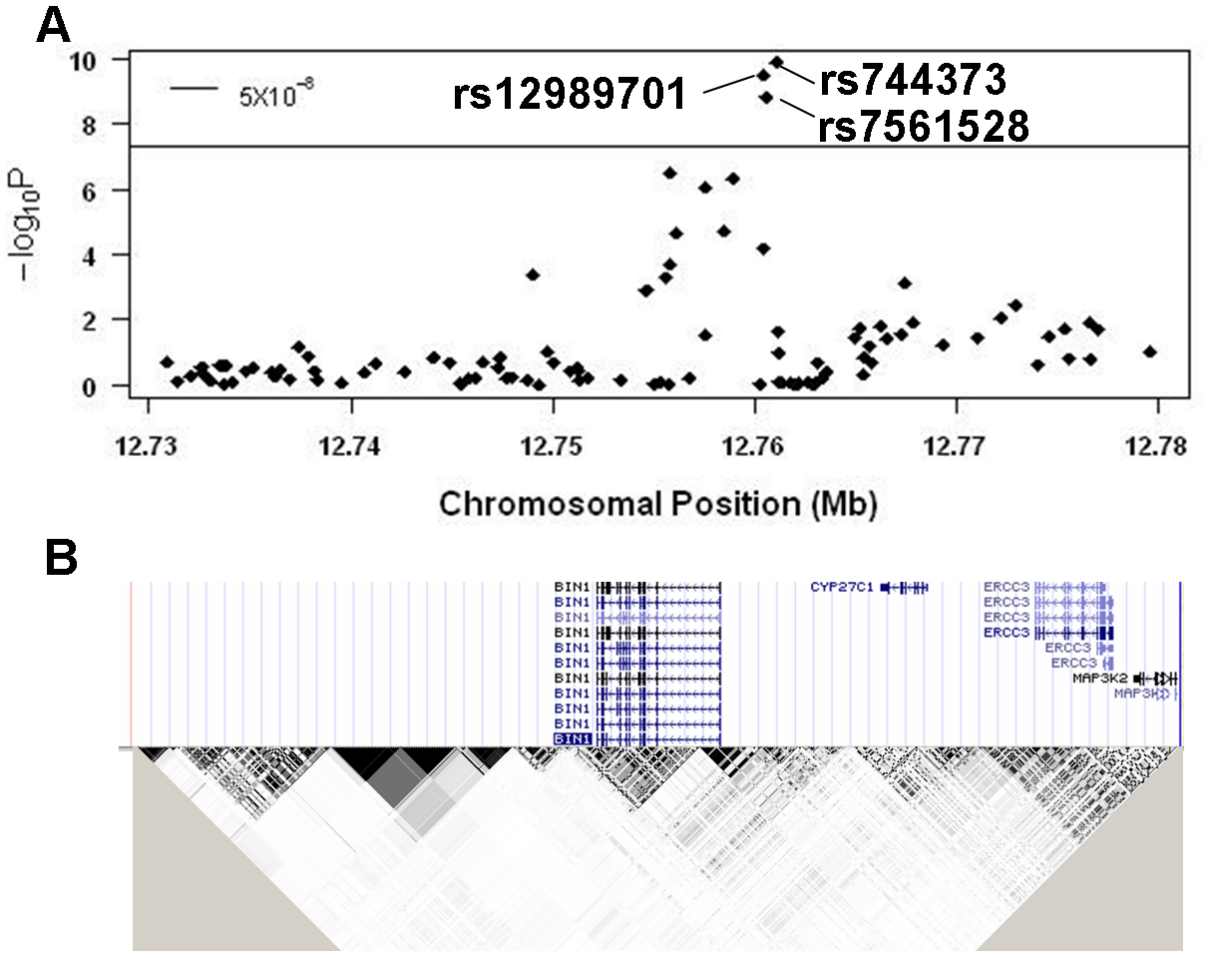
Multiple variants at the *BIN1* locus are strongly associated with AD. A) Meta-analysis for all sample sets (including Pfizer, ADNI, GenADA, Harold and QFP) at the chr2 region (500 kb upstream and downstream of *BIN1*). SNPs rs744373, rs12989701 and rs7561528 are all strongly associated with disease status below the genome-wide significance level. B) Pairwise LD structure (r^2^) calculated in Haploview using HapMap genotype data (phase III) in 60 unrelated CEPH samples (gene structures were shown using the UCSC genome browser for the hg18 assembly). While rs744373 and rs7561528 are in strong LD, limited LD exists between rs12989701 and rs744373 (r^2^ = 0.01 in HapMap samples and r^2^ = 0.05 in Pfizer data set).

We tested the top variants from our GWAS discovery sample set (p<10^−6^) in an independent Genizon set of 751 cases and 751 controls from the Quebec Founder Population (QFP). Besides SNPs adjacent to the APOE locus, we only replicated the SNP (rs12989701) at the *BIN1* locus (p = 0.00216, OR = 1.34). The SNP reached genome-wide significance level in the combined set (Figure 1). We further tested all markers in this region (approximately 500 Kb regions upstream and downstream of *BIN1*) in QFP and combined all available samples/data (Pfizer, ADNI, GenADA, the replication Genizon samples and the published Harold data set) to fine-map this locus. *BIN1* resides across multiple linkage disequilibrium blocks in which linkage disequilibrium (LD) within the block is generally higher than the one between the blocks (Figure 2B). Three strongly associated markers are all located upstream of *BIN1* although other SNPs in high LD with them could extend into the gene region (Figure 2A and unpublished data). Limited LD between these markers and markers located in adjacent genes suggests that this association signal is likely to be more closely related to *BIN1* although the effect could still due to some long-range haplotypes extending further in the region. Interestingly, rs744373 and rs7561528 are in strong LD (r^2^ = 0.745) while the LD between rs744373 and rs12989701 is quite low (r^2^ = 0.05) suggesting independent contributions to disease susceptibility. Both SNPs passed genome-wide significance level in the combined meta-analysis (Table 3).

**Table 3:** Two variants at the BIN1 locus are associated with Alzheimer's disease susceptibility below the genome-wide significance level with limited LD between them.

*SNP*	*A1*	*A2*	*Data Set*	*MAF Case*	*MAF Control*	*P-value*	*OR*
rs744373	G	A	Pfizer	0.313	0.283	6.87E-02	1.16
chr2:127611085			ADNI	.	.	.	.
BIN1 (29.8 kb Upstream)			GenADA^c^	0.336	0.269	4.92E-05	1.37
			Harold/Germany	0.333	0.277	1.45E-03	1.31
			Harold/UK	0.311	0.28	1.84E-04	1.16
			Harold/US	0.301	0.287	4.05E-01	1.07
			Genizon	0.3	0.269	6.04E-02	1.16
			Combined			1.32E-10	1.19
rs12989701	T	G	Pfizer	0.177	0.138	3.72E-03	1.34
chr2:127604455			ADNI	0.188	0.097	8.95E-05	2.16
			GenADA^c^	0.181	0.155	5.26E-02	1.2
BIN1 (23.1 kb Upstream)			Harold/Germany	0.171	0.142	3.72E-02	1.25
			Harold/UK	0.181	0.159	1.12E-03	1.17
			Harold/US	0.165	0.155	4.82E-01	1.08
			Genizon	0.2	0.157	2.16E-03	1.34
			Combined			3.16E-10	1.23

a rs744373 was removed in the ADNI data set during the QC process (snp call rate <99%).

b rs744373 and rs12989701 have limited linkage disequilibrium (r^2^<0.05) between them and either one cannot fully explain the association at this locus.

c GenADA genotype data for the variants were imputed.

### rs744373 and rs12989701 independently contribute to disease susceptibility

We conducted haplotype conditional analysis in our discovery data set (1831 AD cases and 1764 controls) to investigate whether the effect of rs12989701 is indeed independent of the previously identified rs744373. Distributions of rs12989701 alleles are still significantly different between AD cases and controls even after controlling for the rs744373 alleles (P = 0.002). Similar results were observed for rs744373 (P = 0.0059) when controlling for rs12989701. These results showed that the *BIN1* locus contains multiple variants with conditionally independent associations with disease status.

### Pathway Analysis

Our initial analysis for disease susceptibility focused on individual SNPs without considering any potential interactions of multiple variants. The number of potential SNP combinations, however, increases exponentially and becomes impractical for our current GWAS sample size. We hypothesized that multiple variants in genes in the same pathway may jointly contribute to the association with disease status. To test this hypothesis, we employed GenGen, adapted from a pathway analysis tool originally developed to analyze gene expression by adjusting for different gene sizes and the LD between SNPs [17]. We first tested all the pathways collected in BioCarta and the top pathway in the Pfizer sample set is the Gleevec pathway. We further tested the top four pathways (family-wise error rate<0.45) identified from Pfizer set in two independent sample sets: ADNI and GenADA. The Gleevec pathway appears to be significant in all sample sets (Table 4). The DNA repair induced apoptosis pathway was also replicated in the GenADA data set (P = 0.04) but was not significant in the ADNI data set (Table 4).

**Table 4:** Pathway Analysis Results in Three Independent Sample sets^a^.

	Pfizer Sample Set^b^			ADNI Sample Set^b^		GenADA Sample Set^b^	
Pathway	# of Genes with SNPs	Nominal P-value	Family-wise Error Rate	# of Genes with SNPs	Nominal P-value	# of Genes with SNPs	Nominal P-value
Gleevec Pathway	23	0.003	0.17	23	0.028	21	0.04
Links Between Pyk2 and Map Kinases	28	0.006	0.238	28	0.298	24	0.336
Apoptotic Signal in Response to DNA Damage	22	0.005	0.376	22	0.554	22	0.043
Grown Hormone Signal Pathway	28	0.009	0.44	28	0.333	21	0.131

a GenGen was employed in the pathway analysis. Pathways were defined in BioCarta (http://www.biocarta.com/).

b Pfizer and ADNI sample set were obtained by Illumina 550/610 K chips and the GenADA sample set were obtained by Affymetrix.

Non-imputed genotype data were employed in the analysis.

### Disease Progression

It is unknown if any of the recently identified disease loci define different progression profiles for AD patients. We tested four genetic variants that achieved genome-wide significance in association with disease susceptibility (*CLU* = rs11136000, *PICALM* = rs3851179, *CR1* = rs3818361, *BIN1* = rs12989701) for their association with disease progression using CDR-sum of boxes (CDR-SB) measured up to 24 months (rs744373 was removed during the QC process for ADNI since its call rate was less than 99%). Progression analysis was done for 597 AD patients with sufficient CDR-SB data. We used a linear repeated measure mixed model and adjusted for study, age, gender, baseline MMSE, baseline CDR and *APOE e4* status. In AD, baseline MMSE (p<10^−4^) and study (p<0.008) are the only covariates with significant contributions to change of baseline CDR over time. Note that these observations are consistent in all variants tested in our analysis. Among the four markers tested in our data set, only one marker, *PICALM* (rs3851179) showed nominal significant genotype effects on the change in CDR-SB over time for AD subjects (p = 0.02, Bonferroni adjusted p = 0.08), with the TC genotype showing a greater increase than either the TT or CC genotype. The CLU variant showed nominal significant genotype and time interaction (p = 0.02) which would not survive multiple test correction. The other variants are non-significant at the 0.05 level (Table 5).

**Table 5:** AD Progression Analysis for validated variants in AD susceptibility^a^.

*SNP*	*Gene*	*Chr*	*Position*	Genotype*Time Interaction Effect Nominal P value b	*Genotype Effect Nominal P-value* b
rs11136000	*CLU*	8	27520436	0.037	0.966
rs3851179	*PICALM*	11	85546288	0.064	0.021
rs3818361	*CR1*	1	205851591	0.169	0.603
rs744373	*BIN1*	2	127611085	0.548	0.220
rs12989701	*BIN1*	2	127604455	0.725	0.497

a The analysis uses change of CDR-SB as endpoint and a repeated mixed model to adjust for study, age, gender, baseline MMSE, baseline CDR-SB and APOE e4 status (+/−).

b The corrected p-value cutoff after Bonferroni correction is 0.01. None of the variants passed multiple test correction.

## Discussion

Alzheimer's disease has a complex etiology involving interplays of multiple genetic and environmental factors. Despite earlier successes in gene mappings for familial early onset AD cases and identification of the APOE e4 variant for late onset AD cases, the majority of genetic risk involved in LOAD etiology remains largely unexplained. A few robust genetic loci have recently emerged from GWAS studies involving thousands of cases and controls. In this study, we conducted GWAS analysis in an additional 1034 AD/1186 Control subjects and combined this with available data sets to identify and replicate genetic loci related to late-onset AD susceptibility.

We replicated associations with *CR1* and *PICALM* variants in independent samples from the Harold [13] and Lambert studies [14] (Table 2). The *PICALM* variants may be confounded by the *APOE* effects as the association greatly attenuates when we adjust for APOE status. Although we did not replicate the *CLU* variant at the 0.05 significance level, the OR for the variant appears to be consistent in our sample set and this is likely due to the lack of power in the study. The results support the CR1 locus as *bone fide* loci for AD etiology in Caucasians, consistent with the recent studies which replicated *PICALM* and *CLU* loci in independent studies [19]. Different ethnic groups may share the same risk loci such as *SNCA* and *LRRK2* for Parkinson's disease (PD) in Japanese and European cohorts [20], [21] while other loci may show population specificity (e.g. *MAPT* in PD). Future association studies in other ethnic groups may facilitate our understandings of the similarities and differences in the newly identified genetic loci contributing to Alzheimer's disease.

Current disease-modifying strategies for AD therapy have focused on the production and clearance of the amyloid-beta peptide [22]. A solid line of evidence supports the production of amyloid-beta especially the Abeta42 isoform as a primary culprit for the onset of the disease. It was recently shown that the N-terminus of APP may trigger apoptosis [23]. The ongoing clinical trials targeting amyloid-beta are designed to test the critical hypothesis that interference with the A-beta pathway is sufficient to improve cognitive function in AD patients. If the plaque formation induces injury that cannot be easily repaired by removal of the plaques, early intervention is required and additional therapeutic targets will be valuable. New findings from the recent GWAS studies potentially nominate/support additional mechanisms and pathways for the treatment of sporadic late-onset AD patients. The discovery of the *CLU* association underscores the importance of genes involved in lipid metabolism as both *CLU* and *APOE* are related to this process (For a recent review, see [24]). Although prevailing evidences suggest that *APOE e4* is involved in amyloid-beta aggregation and clearance, we cannot rule out other mechanisms such as neuro-inflammation which is also supported by the newly emerged *CR1* locus and *CLU* with a well-established role in inflammation. This is largely consistent with our knowledge from epidemiological studies which identified cardiovascular factors such as midlife high blood pressure, obesity and diabetes with increasing risk of AD while anti-inflammatory drugs seem to reduce risk of dementia. Note that all of the variants identified from the GWAS findings are in non-coding regions and the functional consequences of these variants remain largely unknown, thus follow-up sequencing studies and functional experiments will be required.

Our study further strengthened genetic evidences to support the BIN1 locus which was recently identified in an independent study [25]. Both studies reached genome-wide significance level and there are no known overlaps between the sample sets. Top SNP (rs12989701) in our study is very close to SNP rs744373 in the other study but they are poorly correlated (r^2^<0.05). Both SNPs are replicated in the other study at the 0.05 level but only one reached genome-wide significance level in each individual study. The potential independent contributions of both SNPs were supported by additional haplotype conditional analysis. SNP rs12989701 is located at an evolutionarily conserved region, suggesting that it might be important for gene regulation. *BIN1* (Bridging Integrator 1) was initially identified as a tumor suppressor with a *MYC*-interacting domain, a SH3 domain and a BAR (Bin1 Amphiphysin RVS167) domain [26]. Mutations in *BIN1* were identified in multiple individuals with autosomal recessive centronuclear myopathy [27]. It encodes several alternatively spliced isoforms including brain-specific isoforms [28]. Several *BIN1* isoforms have been shown to associate with dynamin mediated synaptic endocytosis process [29]. Interestingly, endocytosis is also related to *PICALM*, another gene strongly associated with AD. The important role of dynamin mediated endocytosis process was supported by the observations that dynamin-1 levels were reduced in hippocampal neurons in the Tg2576 mouse model of AD [30]. *Amphiphysin 1* knock-out mice lacking *BIN1* expression in the brain and demonstrated deficient endocytic protein scaffolds and synaptic vesicle recycling [31]. Additional evidence from gene knock-outs in Drosophila [32], mice [33] and yeast [34] suggested that *BIN1* may not be essential for endocytosis but may be important for vesicle trafficking [35]. A recent paper demonstrates that *BIN1* is a key component in endocytic endosome recycling in *C. elegans* [36] which suggests a potential role of *BIN1* in endosome function. Endocytic process has been previously implicated in AD as APP, A-beta and ApoE proteins are all internalized through the endolysosomal trafficking pathway. These proteins were further sorted to endosomes. It will be interesting to further investigate the roles of BIN1 in endocytosis/trafficking and its potential contributions to synaptic function.

Most GWAS analysis focused on individual SNPs have a stringent threshold for significance that must be applied due to the number of tests conducted in the study. It is possible that multiple variants can jointly contribute to disease status. We therefore conducted pathway analysis which derived an enrichment score for all genes in a pathway and compared this with the distribution under null hypothesis based on random permutation. This analysis adjusts for differences in gene sizes and maintains the correlation structures among the SNPs. The apoptotic signal induced by DNA damage has an enriched distribution that significantly deviates from the null in both the Pfizer and GenADA sample sets. Interestingly, our unbiased scan based on pathways collected in Biocarta also indicated that the overall distribution for all the SNPs within the downstream genes targeted by Gleevec appears to be significantly different from the null distribution. Although none of the loci appear to be genome-wide significant, combinations of these SNPs provide evidence to support the involvement of the pathway. Gleevec, a cancer drug approved for the treatment of chronic myeloid leukemia, was recently shown to reduce gamma-secretase cleavage for APP [37]. One recent study suggests that Gleevec can bind to a gamma-secretase modulator [38]. Our results, if further validated, may provide additional insights about the potential mechanism of Gleevec in Alzheimer's disease.

We examined the association of the robust disease susceptibility loci in 597 AD patients with sufficient longitudinal clinical data. We observed that the *e4* allele in *APOE* was not associated with progression in AD patients although it was shown to be significantly associated with a faster rate of progression in MCI patients in the previous study [18]. AD patients with heterozygous genotype at the *PICALM* variant rs3851179 have a faster rate of progression compared with CC carriers. The rate of progression in the TT genotypes has a slight increase compared with CC carriers although far from statistical significance. We also observed that the variant at *CLU* has a nominal significant interaction with time. All the effects from *PICALM* and *CLU* variants are independent of the known risk factors such as *APOE e4* allele, age and baseline MMSE scores but do not pass multiple test correction so it likely still represents a false positive signal. Our results indicated that the recently identified variants for AD susceptibility may have limited utility to predict disease progression in AD patients. Further unbiased GWAS studies using disease progression as endpoints may be fruitful if statistical power becomes sufficient. Follow up deep sequencing studies and functional experiments for these genetic loci may increase our understanding of the disease mechanisms for AD.

## Methods

### Subjects

The Pfizer sample collection includes a total of 1034 cases and 1186 controls: 489 subjects from the Lipitor's Effect in Alzheimer's Dementia (LEADe) trial [39]–[40] , 180 MCI subjects from the Vitamin E trial who have converted to AD during the course of the study [18], 216 probable AD subjects enrolled by PrecisionMed for case/control study and 149 subjects from clinical trial A3041005 which is a phase II trial investigating CP-457920 (a selective alpha5 GABAA receptor inverse agonist) in Alzheimer's disease. Samples were collected from multiple clinical sites, and the ethics committees with jurisdiction over these sites each gave approval for future research including that represented by the work in this paper. Written informed consent was given by the subjects for their information to be stored in the database and used for the research described in this paper. All subjects were diagnosed with probable or possible AD if they met NINCDS and/or DSM-IV criteria and had mini-mental state examination (MMSE) scores below 25 at baseline. The control subjects included 234 subjects from PrecisionMed for case/control study, 883 subjects from A9010012 which is a method study to collect elderly subjects free of any neurological and psychiatric conditions, and 69 subjects from 999-GEN-0583-001 which is another method study to obtain DNA in a reference population of Caucasians defined as psychiatric and neurological normal. Controls have no neuropsychiatric diseases and their MMSE scores were above 27 at the time of enrollment. For AD susceptibility analysis, we removed any potential early-onset AD cases (age of onset less than 65). All the controls were re-matched with the remaining cases according to gender, age (controls are older than the cases) and ethnicity (only Caucasians were selected in the analysis). The final Pfizer GWAS analysis set for AD susceptibility contains 733 LOAD cases and 792 controls. ADNI is a large three-year study with the primary objective of identifying biomarkers of Alzheimer's disease through multiple technology platforms including genetics and neuroimaging. Genotype data were generated from approximately 800 subjects through the Illumina 610Quad platform (http://www.loni.ucla.edu/ADNI/Data/). 300 AD subjects (including MCI subjects who had converted to AD) and 196 controls from ADNI were included in the analysis. Clinical information for these subjects was described previously [11], [41]. The GenADA sample set contains 801 patients that met the NINCDS-ADRAD and DSM-IV criteria for probable AD and 776 control subjects with no history of dementia [8] (http://www.ncbi.nlm.nih.gov/gap). 798 AD subjects from the GenADA collection were included in the analysis after completion of QC procedures. In total, our GWAS discovery analysis set for AD susceptibility comprises of 1831 AD cases and 1764 controls from Pfizer, ADNI and GenADA. The ADNI and GenADA studies were selected based on their sample size and availability at the time of the study. Among the 685 AD subjects who have longitudinal clinical data, 161 subjects from ADNI and 436 subjects from LEADe with sufficient CDR-SB data were included in the disease progression analysis.

### The Genizon Sample Set

1502 samples from the Quebec Founder Population (QFP) were included in the study as a replication set (case/control ratio = 1). All Alzheimer's disease subjects were 65 years old or older and presented with probable AD based on DSM-IV criteria or definite AD as confirmed by neuropathology findings on autopsy. The controls were matched to the patients for gender. The controls were 75 years and older and were absent of AD based on a Mini-Mental State Examination (MMSE) score test> = 26 (adjusted for age and education) and a Montreal Cognitive Assessment (MoCA) score test> = 26 (adjusted for education) at the time of recruitment.

### Genotyping

All genomic DNA samples for Pfizer and Genizon were extracted from blood and quantified using Picogreen (Invitrogen Inc). The first batch of Pfizer samples (∼300 cases from PrecisionMed/A3041005 and matched controls plus 489 cases from LEADe) were processed with the Illumina HumanHap550 array while all remaining samples were genotyped using the Illumina 610Quad array. All genotyping was performed at Genizon Biosciences Inc and genotype calls were generated after clustering all the data within each platform. Most of LEADe samples were processed on both 550 and 610 platforms and the genotype data concordance rates were greater than 99.99%. The ADNI genetic data set was downloaded from the ADNI web site and a similar initial QC process was performed at Pfizer (the final data set after QC includes 509376 markers in 719 subjects). The GenADA data was downloaded from dbGap and the data were imputed based on the reference haplotypes from Hapmap III using Mach [42]–[43]. Genotype data from the Genizon samples were obtained from Illumina HumanHap 550 array.

### Genotype data Quality Control

Data cleaning and Quality control were performed with PLINK using the identical criteria for all Pfizer, ADNI and Genizon sample sets obtained from Illumina platforms. SNPs with MAF <1% or more than 1% missing values were removed, as were samples with more than 1% missing values. Hardy-Weinberg equilibrium (HWE) was evaluated in the control population. SNPs that were out of HWE (−log (p)>5) were dropped. Sample sets were checked for genetic outliers and duplicated samples, which were removed. Only one of any group of samples that are strongly related (IBS distance <0.1) was kept. Reported gender was cross-checked with genetic gender to identify any possible sample identification errors. SNPs with an excess of heterozygosity were removed (Het Excess>0.1 and HWE p<0.01). Caucasians were identified based on multi-dimensional scaling (MDS) of the data compare to the CEPH samples in the HapMap dataset. We adapted the QC procedure from the original GenADA set to accommodate the Affymetrix 550 k platform [8]. We removed three additional subjects from the analysis set (subject ID 781, 6145 and 2803) who appear to be either admixture or more distant from the cluster formed by the other Caucasian subjects in the population stratification analysis.

### Imputation

GenADA genotype data (after QC) were imputed using Mach (http://www.sph.umich.edu/csg/abecasis/mach/, [42] [43]) based on reference haplotypes from HapMap III phased data (release 2). We performed two-step imputation as recommended for large scale studies: the first step to calibrate model parameters and the second step to impute actual genotypes. Variants with poor imputation quality scores (r^2^ less than 0.3) and minor allele frequency less than 1% were removed after imputation.

### Statistical Analysis for Disease Susceptibility

We performed case/control allelic chi-square tests in Pfizer, ADNI and GenADA sample set separately using PLINK (http://pngu.mgh.harvard.edu/purcell/plink/). We checked the alleles in the association files to ensure that they are consistent across all data sets. The inflation factor, lambda, was estimated by dividing the median chi-square values by 0.455 (the expected value under the null hypothesis) for each data set. The resulting p-values were combined across datasets using a weighted z-score approach [16]. We calculated association test results from the published Harold study based on genotype counts in cases and controls from each individual cohort (US, UK and Germany). In the replication study, we analyzed additional genotype data for 104 markers from the Genizon samples. To refine the association signal at the *BIN1* locus, we combined association test results from all studies (Pfizer, ADNI, GenADA, Harold US, Harold Germany, Harold UK, and QFP) across the 500 Kb regions upstream and downstream of *BIN1* using the meta-analysis function in PLINK assuming a fixed effect model. To test whether SNPs in this region has contribution to disease susceptibility independent of each other, we performed conditional haplotype analysis using PLINK through comparing the alleles/haplotypes that have a similar haplotype background as defined by the SNP of interest.

### Statistical Analysis for Disease progression

Disease progression was characterized using the Clinical Dementia Rating-Sum of boxes (CDR-SB) score. Longitudinal data were available for 685 AD patients but only 597 subjects with sufficient CDR-SD data up to 24 months are included in the analysis. The genotypic effect of a variant on the change over time in the CDR sum of boxes was assessed using a repeated measures mixed model, with covariates of baseline CDR sum of boxes, baseline MMSE, sex, age at baseline and APOE4 status, with genotype and the genotype*time interaction as the factors of primary interest. A main-effects model, without the genotype*time interaction, was also fit to the data. Progression effects were modeled for four SNPS: *CLU* = rs11136000, *PICALM* = rs3851179, *CR1* = rs3818361, *BIN1* = rs12989701. The other *BIN1* variant rs744373 was not tested since it was removed from the ADNI data set during the QC process.

### Pathway Analysis

The current GWAS analysis is based on association tests in individual markers without considering the joint effects of multiple variants. We employed GenGen [17] to test whether the distribution of statistics from a group of genes in each pathway from BioCarta (http://www.biocarta.com/) is consistently deviated from the null hypothesis from our sample sets. Pfizer, ADNI and GenADA dataset (before imputation) were used for this analysis. 1000 permutations were conducted for each analysis.

## Supporting Information

Table S1 Summary statistics for all markers in Pfizer sample set. Note: Large file (41MB).
(XLSX)
